# Administration of anti-receptor activator of nuclear factor-kappa B ligand (RANKL) antibody for the treatment of osteoporosis was associated with amelioration of hepatitis in a female patient with growth hormone deficiency: a case report

**DOI:** 10.1186/s12902-016-0148-0

**Published:** 2016-11-24

**Authors:** Ayumu Takeno, Masahiro Yamamoto, Masakazu Notsu, Toshitsugu Sugimoto

**Affiliations:** Internal Medicine 1, Shimane University Faculty of Medicine, 89-1, Enya-cho, Izumo, Shimane 693-8501 Japan

**Keywords:** Growth hormone deficiency (GHD), Non-alcoholic fatty liver disease (NAFLD), Non-alcoholic steatohepatitis (NASH), Receptor activator of nuclear factor-kappa B ligand (RANKL), Denosumab

## Abstract

**Background:**

Growth hormone deficiency (GHD) is associated with non-alcoholic fatty liver disease (NAFLD). A recent animal study showed that hepatocyte-specific receptor activator of nuclear factor-kappa B (RANK) knockout mice had significantly lower liver fat content compared with control mice concomitant with a decrease in production of inflammatory cytokines such as tumor necrosis factor-α (TNF-α) from hepatocytes and kupffer cells. The role of anti-RANK ligand (RANKL) antibody for osteoporosis on hepatitis in patients with aGHD is still unknown.

**Case presentation:**

A forty-seven-year-old female patient was referred to our hospital to investigate chronic hepatitis caused by unknown etiology. She had past history of craniopharyngioma treated with craniotomy and post-surgical radiotherapy. She was for the first time diagnosed as panhypopituitarism including growth hormone deficiency and osteoporosis by endocrine examinations and bone mineral densitometry, respectively. In addition, non-alcoholic steatohepatitis (NASH) was histologically confirmed by liver biopsy in this time. Sixty mg anti-RANKL antibody, which was subcutaneously injected to treat the osteoporosis every six months after replacement of 5 mg hydrocortisone and 30 μg oral desmopressin, rapidly decreased the levels of her liver enzymes (ALT and γGTP were 133 to 72 U/L and 284 to 99 U/L at 16 months after the beginning of the treatment, respectively). Additional amelioration of liver dysfunction was not observed after growth hormone replacement.

**Conclusions:**

The clinical course of the present case suggested that RANKL-RANK signaling may be a key pathological mechanism in establishment or development of NAFLD or NASH in patients with panhypopituitarism including GHD.

## Background

Non-alcoholic fatty liver disease (NAFLD) defines liver abnormalities ranging from simple steatosis to non-alcoholic steatohepatitis (NASH) with or without cirrhosis development. Prevalence of NAFLD is massively increasing because that is related to obesity [1]. Recently, several clinical studies have shown that growth hormone deficiency (GHD) is complicated with NAFLD [2, 3] and that growth hormone (GH) replacement therapy improves liver dysfunction [4–6] and histological findings of NAFLD [4, 6], suggesting that GHD is considered as one of the pathological causes for NAFLD. However, detailed mechanisms of establishment of NAFLD in patients with GHD are still unknown.

Receptor activator of nuclear factor-kappa B ligand (RANKL) is an osteoclast differentiating factor which binds to receptor activator of nuclear factor-kappa B (RANK) on the surface of osteoclasts and enhances osteoclast differentiation, function and bone resorption [7]. An anti-RANKL antibody, denosumab, is clinically used as an anti-osteoporotic agent which increases bone mineral density (BMD) by inhibiting bone resorption [8, 9]. We report the first case of adult-GHD (aGHD) that administration of anti-RANKL antibody for the treatment of osteoporosis was associated with amelioration of hepatitis.

## Case presentation

A 47-year-old debile female patient was referred to our hospital to examine hepatitis caused by unknown etiology. At 11 years of age, she was diagnosed as craniopharyngioma and treated with craniotomy and post-surgical radiotherapy. Sex hormone replacement was performed for hypogonadotropic hypogonadism from 15 to 44 years old. Elevated liver enzymes were observed about past 20 years.

**Fig. 1:**
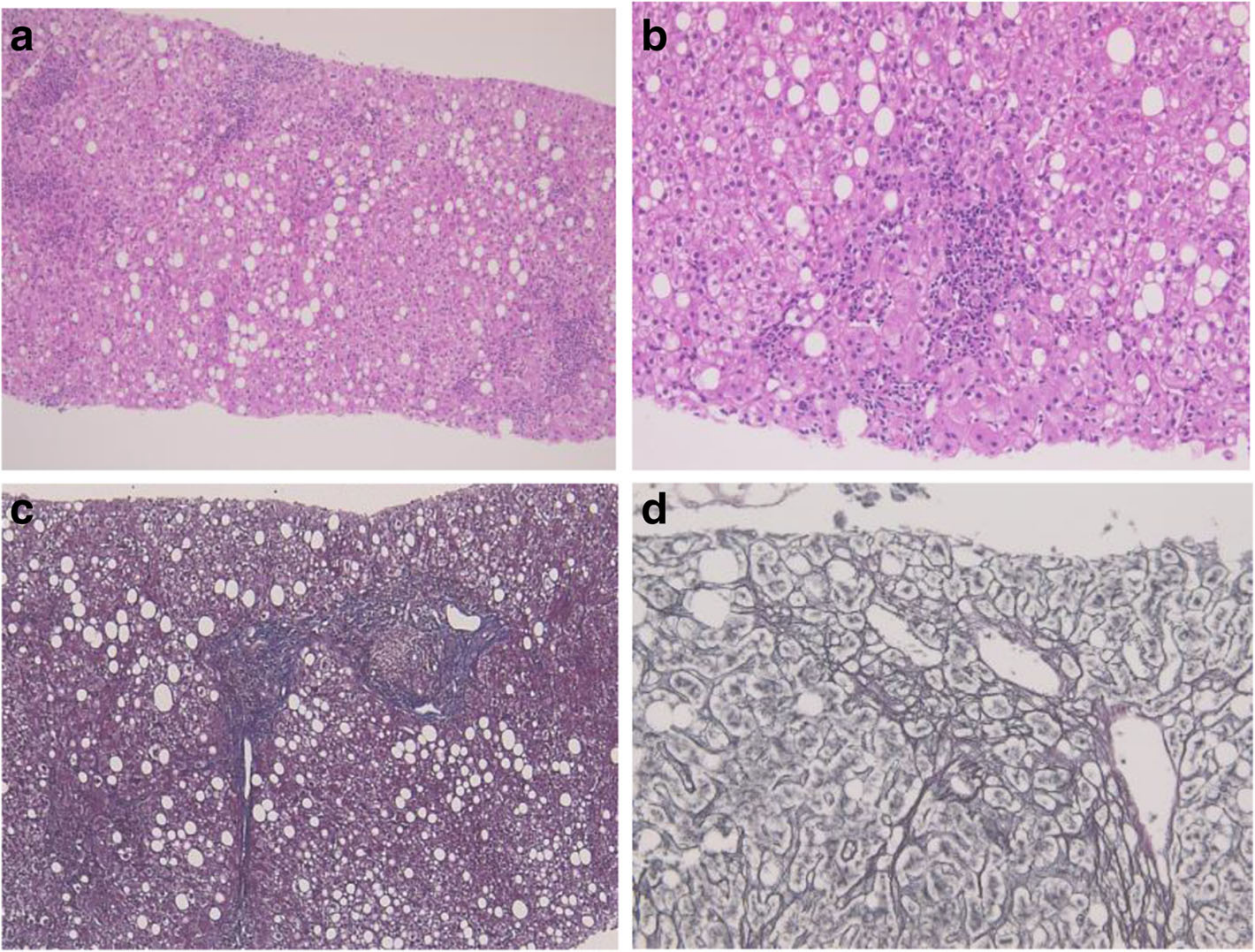
Liver biopsy samples. Hematoxylin and eosin (H&E) staining showed steatosis (50%), infiltrations of inflammatory cells and hepatocyte ballooning (Fig. 1**a** and **b**: ×100 and ×400, respectively). Azan-mallory staining showed fibrosis of portal region (Fig. 1**c**: ×100), and silver staining showed pericellular fibrosis (Fig. 1**d**: ×400). These findings were compatible to the diagnosis of NASH. These findings were compatible with NASH (grade 2 and stage 2)

She was an underweight woman (body height, 149.3 cm; body weight, 36.5 kg; BMI 16.4 kg/m^2^) who had no alcohol consumption. Laboratory findings showed elevated liver enzymes (AST, ALT and γGTP were 131 U/L, 106 U/L and 238 U/L, respectively) concomitant with an extremely low concentration of insulin-like growth factor-1 (IGF-1) (12 ng/mL) (Table 1). Abdominal ultrasonography showed fatty liver. Hepatitis B surface antigen, anti-hepatitis C virus antibody, anti-nuclear antibody and anti-mitochondrial M2 antibody were undetectable. Histological findings of the liver biopsy were compatible with NASH (Fig. 1a–d).

**Table 1:** Laboratory tests

	Level measured		Reference range		
《Biochemical Profiles》					
AST	131 U/L		(10–38)		
ALT	106 U/L		(5–40)		
γGTP	238 U/L		(0–75)		
ALP	439 U/L		(21–80)		
BUN	7.4 mg/dL		(8.0–21.0)		
Cr	0.53 mg/dL		(0.44–0.83)		
Na	145 mEq/L		(137–146)		
K	3.5 mEq/L		(3.5–4.9)		
Cl	111 mEq/L		(98–109)		
LDL-C	98 mg/dL		(70–139)		
HDL-C	38 mg/dL		(40–80)		
TG	106 mg/dL		(50–140)		
HbA1c	5.7%		(4.7–6.2)		
FPG	78 mg/dL		(60–109)		
《Endocrine Profiles》					
GH	0.1 ng/mL		(<3.0)		
IGF-1	12 ng/mL		(83–221)		
PRL	13.3 ng/mL		(3.2–26.2)		
LH	<0.2 mIU/mL		(1.13–88.33)		
FSH	<0.1 mIU/mL		(3.01–16.60)		
E2	<7 pg/mL		(7–509)		
TSH	4.76 μU/mL		(0.50–3.00)		
FT4	0.6 ng/dL		(0.8–1.5)		
FT3	2.4 pg/mL		(2.1–3.8)		
ACTH	17.4 pg/mL		(7.2–63.3)		
cortisol	2 μg/dL		(2–18)		
u-cortisol	undetectable		(11.2–80.3)		
ADH	<0.8 pg/mL		(<4.2)		
s-Osm	289 mOsm/L		(270–295)		
u-Osm	250 L mOsm/L		(50–1300)		
GHRP-2 loading test					
Time (min)	0	15	30	45	60
GH (ng/mL)	0.1	0.4	0.4	0.3	0.2

aGHD was diagnosed by loading test with 100 μg GH-releasing peptide-2 (GHRP-2) [basal level: 0.1 ng/mL, peak level: 0.4 ng/mL at 15 min]. She was diagnosed as ACTH, TSH, LH and FSH insufficiency as well as central diabetes insipidus by additional loading tests. Osteoporosis was diagnosed by BMD [T-score of the lumbar spine and the femoral neck were −2.0 SD and −2.5 SD, respectively].

**Fig. 2:**
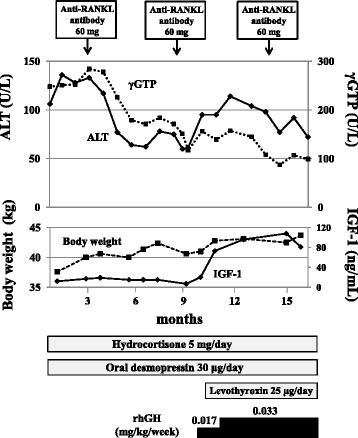
Changes in the liver enzymes, body weight and IGF-1 after the initiation of hormone replacement and anti-RANKL antibody. After the replacement of hydrocortisone 5 mg/day and oral desmopressin 30 μg/day, the liver enzymes were unchanged. Three months later anti-RANKL antibody 60 mg was administered, and plasma levels of ALT and γGTP were decreased from 133 to 62 U/L and from 284 to 171 U/L, respectively. Reduction of liver enzymes was also confirmed after second and third administration of anti-RANKL antibody. GH and levothyroxin replacement were initiated between the second and the third administration of anti-RANKL antibody, and IGF-1 level was increased to normal range. However, the liver enzymes were not decreased by replacement with GH and levothyroxin. She gained 6.1 kg of weight during the treatment period

The therapeutic course was shown in Fig. 2. The levels of liver enzymes did not change for three months after the replacement of hydrocortisone (5 mg/day) and oral desmopressin (30 μg/day). When 60 mg anti-RANKL antibody was subcutaneously injected to treat the osteoporosis every six months, the levels of her liver enzymes were rapidly decreased after the first and second administration (levels of ALT and γGTP at nine months later were 60 U/L and 117 U/L, respectively). Then, GH replacement therapy (0.033 mg/kg/week after 0.017 mg/kg/week for two month) and levothyroxine (25 μg/day) were initiated, however, the levels of liver enzymes were increased and were not changed despite improvement of IGF-1 concentrations (ALT and γGTP were 114 U/L and 157 U/L at around 12 months after the beginning of the treatment, respectively). However, the levels of liver enzymes were decreased again after third administration of anti-RANKL antibody (ALT and γGTP were 72 U/L and 99 U/L at 16 months after the beginning of the treatment, respectively).

## Discussion

NASH is characterized by inflammation and fibrosis in addition to fat deposition within hepatocytes. Many pro-inflammatory cytokines including tumor necrosis factor-α (TNF-α) are involved in the progression of NASH [10–12]. A recent study showed that hepatocyte-specific RANK knockout mice had significantly lower liver fat content compared with control mice concomitant with a decrease in production of inflammatory cytokines such as TNF-α from hepatocytes and kupffer cells [13], indicating that RANKL-RANK signaling pathway potentially is associated with hepatitis. In our case, the amelioration of liver enzymes was associated with the administration of anti-RANKL antibody, suggesting that RANKL-RANK signaling pathway may be one of the potential pathogenesis for the development of NAFLD or NASH under the condition of panhypopituitarism including GH deficiency.

Unlike previous cases [4–6], GH replacement therapy was not clearly associated with liver dysfunction in this case. Sufficient dose of GH was given to her because her IGF-1 levels were within normal range for her age. Matsumoto et al. reported that improvement of liver dysfunction after GH replacement in GHD patients was observed at least within three months [5], indicating that short duration of the GH replacement was not the reason for prolonged liver dysfunction in this case. In contrast to previous cases, GH was replaced under the administration of anti-RANKL antibody in this case. The finding that GH replacement after administration of anti-RANKL antibody was not effective suggested that mechanism of amelioration of hepatitis by GH replacement therapy may be identical with inhibition of RANK signaling for production of inflammatory cytokines in hepatocytes. This observation indicated that RANKL-RANK signaling may play a crucial role in progression of NAFLD or NASH in patients with panhypopituitarism including GH deficiency.

## Conclusions

Present case showed that administration of anti-RANKL antibody for the treatment of osteoporosis was associated with amelioration of hepatitis in a women patient with aGHD concomitant with NASH. The clinical course of the present case suggested that RANKL-RANK signaling may be a key pathological mechanism in development of NASH in patients with panhypopituitarism including GH deficiency. Agents to inhibit RANK-RANKL signaling in hepatocytes might be beneficial for treatment of NASH in these populations.

## Abbreviations

aGHD: Adult growth hormone deficiency
BMD: Bone mineral density
GHD: Growth hormone deficiency
GHRP-2: Growth hormone releasing peptide-2
IGF-1: Insulin-like growth factor-1
NAFLD: Non-alcoholic fatty liver disease
NASH: Non-alcoholic steatohepatitis
RANK: Receptor activator of nuclear factor-kappa B
RANKL: Receptor activator of nuclear factor-kappa B ligand
T2DM: Type 2 diabetes mellitus
